# Exploitation of Scavenger Receptor, Macrophage Receptor with Collagenous Structure, by *Cryptococcus neoformans* Promotes Alternative Activation of Pulmonary Lymph Node CD11b^+^ Conventional Dendritic Cells and Non-Protective Th2 Bias

**DOI:** 10.3389/fimmu.2017.01231

**Published:** 2017-09-28

**Authors:** Jintao Xu, Adam Flaczyk, Lori M. Neal, Zhenzong Fa, Daphne Cheng, Mike Ivey, Bethany B. Moore, Jeffrey L. Curtis, John J. Osterholzer, Michal A. Olszewski

**Affiliations:** ^1^Division of Pulmonary and Critical Care Medicine, Department of Internal Medicine, University of Michigan Health System, Ann Arbor, MI, United States; ^2^Department of Veterans Affairs Health System, VA Ann Arbor Healthcare System (VHA), Ann Arbor, MI, United States; ^3^Department of Microbiology and Immunology, University of Michigan Medical School, Ann Arbor, MI, United States

**Keywords:** scavenger receptor macrophage receptor with collagenous structure, fungal persistence, Th2 response, CD11b^+^ conventional DC, *Cryptococcus neoformans*

## Abstract

Macrophage receptor with collagenous structure (MARCO) contributes to fungal containment during the early/innate phase of cryptococcal infection; however, its role in adaptive antifungal immunity remains unknown. Using a murine model of cryptococcosis, we compared host adaptive immune responses in wild-type and MARCO^−/−^ mice throughout an extended time course post-infection. Unlike in early infection, MARCO deficiency resulted in improved pulmonary fungal clearance and diminished cryptococcal dissemination during the efferent phase. Improved fungal control in the absence of MARCO expression was associated with enhanced hallmarks of protective Th1-immunity, including higher frequency of pulmonary TNF-α-producing T cells, increased cryptococcal-antigen-triggered IFN-γ and TNF-α production by splenocytes, and enhanced expression of M1 polarization genes by pulmonary macrophages. Concurrently, we found lower frequencies of IL-5- and IL-13-producing T cells in the lungs, impaired production of IL-4 and IL-10 by cryptococcal antigen-pulsed splenocytes, and diminished serum IgE, which were hallmarks of profoundly suppressed efferent Th2 responses in MARCO-deficient mice compared to WT mice. Mechanistically, we found that MARCO expression facilitated early accumulation and alternative activation of CD11b^+^ conventional DC (cDC) in the lung-associated lymph nodes (LALNs), which contributed to the progressive shift of the immune response from Th1 toward Th2 at the priming site (LALNs) and local infection site (lungs) during the efferent phase of cryptococcal infection. Taken together, our study shows that MARCO can be exploited by the fungal pathogen to promote accumulation and alternative activation of CD11b^+^ cDC in the LALN, which in turn alters Th1/Th2 balance to promote fungal persistence and dissemination.

## Introduction

*Cryptococcus neoformans* is an environmental fungal pathogen that causes severe meningoencephalitis with mortality rates of up to 20–60%, despite the availability of antifungal drugs (1). Cryptococcal infections generally manifest in patients compromised due to HIV infection, cancer, or organ transplantation. However, up to 25% of cases reported in the United States and 60% of cases in China occur in patients without recognizable immune-deficiencies (2, 3). Furthermore, Cryptococci can persist within immune competent hosts for an extended period of time and can reactivate following immune-compromise (4). Increasing evidence suggests that both host immune dysfunction and immune modulation induced by *C. neoformans* contributes to fungal persistence (2, 5–8).

One major way through which *C. neoformans* promotes fungal persistence is by altering CD4^+^ helper T (Th) cell polarization and subsequent macrophage activation status (9, 10). Virulence factors that contribute to the shift of host responses away from protective Th1 to non-protective Th2 bias correlate with invasive fungal disease severity [reviewed in Ref. (2, 11)]. The absence of protective Th1 cytokines (IFN-γ and TNF-α) and enhanced production of Th2 type cytokines (IL-4, IL-5, and IL-13) facilitate alternative activation of macrophages, which enable intracellular survival and growth of *C. neoformans* (12, 13). Th cell differentiation is orchestrated by antigen-presenting cells. During *C. neoformans* infection, pulmonary CD11b^+^ conventional DC (cDC) are the primary cellular population responsible for non-protective Th2 cell polarization (14, 15). However, the factors that modulate the migration and activation of CD11b^+^ cDC during fungal infection are not well understood. Although skewing of Th2 responses is one well-established effect of cryptococcal virulence factors (6–8, 12, 15), very few loopholes in the host immune system that can be exploited by *C. neoformans* to promote non-protective responses have been identified (15–17). Identification of host factors that contribute to non-protective responses and enable fungal persistence may reveal new targets for promoting effective T cell responses that are crucial for combat fungal infections.

Macrophage receptor with collagenous structure (MARCO) (human Gene ID: 8685) is a scavenger receptor that plays important roles in host defense against viral, bacterial, and parasitic infections (18). Our recent study showed that MARCO expression contributes to fungal containment during the innate phase of cryptococcal infection by enhancing production of pro-inflammatory cytokines and recruitment of mononuclear phagocytes to the infection sites (19). Besides its well-studied roles in antibacterial and antifungal innate immunity (20–24), the importance of MARCO in regulating adaptive immunity is being increasingly recognized. Notably, MARCO expression has been shown to regulate morphology, gene expression, and migration of dendritic cell (DC) upon stimulation; thus, MARCO may act as an important link for the initiation and polarization of adaptive responses (25–27). Recent studies support this paradigm and showed that MARCO is involved in the development of T cell-mediated immunity during tumor inflammation and allergic airway responses (28, 29). While the role of MARCO in modulation of adaptive immunity to fungi remains unclear, a previous study by our group demonstrated that another scavenger receptor, scavenger receptor-A (SR-A), can be exploited by *C. neoformans* to support Th2 immune polarization during infection (17). Thus, we hypothesized that MARCO also regulates T cell-mediated immunity during fungal infections, possibly through modulation of DC migration and/or activation.

In the current study, we focused on the roles of MARCO in the adaptive immune response during *C. neoformans* infection. Using a murine model of cryptococcosis, we found that MARCO expression shifts both local and systemic Th responses away from protective Th1 toward non-protective Th2 response with impaired classical activation of macrophages. Thus, MARCO expression contributes to fungal growth and dissemination during the efferent phase of cryptococcal infection. Mechanistically, we show that MARCO expression facilitates the accumulation and alternative activation of CD11b^+^ cDC in the lung-associated lymph nodes (LALN) which promote the priming of a non-protective Th2 response in secondary lymphoid organs during *C. neoformans* infection.

## Materials and Methods

### Mice

C57BL/6 mice (wild type) were obtained from Jackson Laboratories (Bar Harbor, ME, USA). MARCO-deficient (MARCO^−/−^) mice, obtained initially from Dr. Lester Kobzik (21), were bred and housed under specific pathogen-free conditions in the Animal Care Facility at the VA Ann Arbor Healthcare System. Both male and female mice were between the ages of 6–12 week at the time of infection and were humanely euthanized by CO_2_ inhalation at the time of data collection. All experiments were approved by the Institutional Animal Committee on Use and Care and the Veterans Administration Institutional Animal Care and Use Committee under protocol # 0512-025 and were carried out according to NIH guidelines and the Guide for the Care and Use of Laboratory Animals.

### *Cryptococcus* *neoformans*

*Cryptococcus neoformans* strain 52D was recovered from 10% glycerol frozen stocks stored at −80°C and grown to stationary phase at 37°C using Sabouraud dextrose broth (1% Neopeptone, 2% dextrose; Difco, Detroit, MI, USA) on a shaker. The cultures were centrifuged and washed with non-pyrogenic saline (Travenol, Deerfield, IL, USA). Cells were counted *via* hemocytometer and diluted to 3.3 × 10^5^ yeast/ml in sterile non-pyrogenic saline.

### Intratracheal Inoculation of *C. neoformans*

Mice were anesthetized *via* intraperitoneal (i.p.) injection of ketamine (100 mg/kg body weight) plus xylazine (6.8 mg/kg) and were restrained on a foam plate. A small incision was made through the skin covering the trachea. The underlying salivary glands and muscles were separated. A 30-G needle was attached to a 1 ml tuberculin syringe with *C. neoformans* suspension (3.3 × 10^5^ yeast/ml) and infection was performed by intratracheally injecting 30 µl (10^4^ CFU) of inoculum into the lungs. After inoculation, the skin was closed with cyanoacrylate adhesive and the mice were monitored during recovery from the anesthesia.

### Lung Leukocyte Isolation

The lungs from each mouse were excised, washed in RPMI 1640 and digested enzymatically as previously described (30). In brief, lungs were minced with scissors followed by Gentle MACS homogenization and incubated at 37°C for 35 min in 5 ml/mouse digestion buffer [RPMI 1640, 5% FBS, penicillin, and streptomycin (Invitrogen, Grand Island, NY, USA); 1 mg/ml collagenase A (Roche Diagnostics, Indianapolis, IN, USA); and 30 µg/ml DNase I]. The cell suspension and tissue fragments were further dispersed by Gentle MACS homogenization and were centrifuged. Erythrocytes in the cell pellets were lysed by addition of 3 ml NH_4_Cl buffer (0.829% NH_4_Cl, 0.1% KHCO_3_, and 0.0372% Na_2_EDTA, pH 7.4) for 3 min followed by a threefold excess of RPMI 1640. Cells were re-suspended and subjected to syringe dispersion and filtered through a sterile 100-µm nylon screen (Nitex, Kansas City, MO, USA). The filtrate was centrifuged for 30 min at 1,500 × *g* with no brake in the presence of 20% Percoll (Sigma) to separate leukocytes from cell debris and epithelial cells. Leukocyte pellets were re-suspended in complete RPMI 1640 media and enumerated on a hemocytometer after dilution in trypan blue (Sigma).

### CFU Assay

For determination of fungal burden, small aliquots of digested lungs, spleens, and brains were collected. Series of 10-fold dilutions of the samples were plated on Sabouraud dextrose agar plates in duplicate 10-µl aliquots and incubated at room temperature. *C. neoformans* colonies were counted 2 days later and the number of CFU was calculated on a per-organ basis.

### LALN Leukocyte Isolation

Individual LALN were excised and then dispersed using a 1-ml sterile syringe plunger and flushed through a 70-µm cell strainer (BD Falcon, Bedford, MA, USA) with complete media into a sterile tube. After being centrifuged at 2,500 rpm/min for 5 min, the supernatant was removed and the cell pellets were used for gene expression analysis by quantitative real-time RT-PCR (qRT-PCR) or flow cytometry.

### Ag-Specific Cytokine Production by Splenocytes

Spleens were excised and dispersed using a 3-ml sterile syringe plunger and flushed through a 70-µm cell strainer (BD Falcon) with complete media. Isolated spleen cells were cultured in media with heat-killed *C. neoformans* in a ratio of 1:2 in 6-well plates with 2 ml complete RPMI 1640 medium at 37°C and 5% CO2 for 48 h. Supernatants were stored and analyzed for cytokine levels as described earlier. The Ag-specific cytokine production was quantified using a LEGENDplex cytometric bead array (CBA) kit (BioLegend, San Diego, CA, USA) following the manufacturer’s specifications and read on an LSRII flow cytometer (Becton, Dickinson Immunocytometry Systems, Mountain View, CA, USA). Analysis was performed using BioLegend’s LEGENDplex software.

### Total Serum IgE

Serum was obtained from the blood samples collected by severing the vena cava of the mice before lung excision. Blood samples were then allowed to clot and were spun to separate serum. Serum samples were diluted 100-fold and assayed for total IgE levels using a mouse IgE sandwich ELISA kit (BioLegend, San Diego, CA, USA) following the manufacturer’s specifications.

### RT-qPCR

Total RNA from lung leukocytes was prepared using TRIzol reagent (Invitrogen), and first-strand cDNA was synthesized using Reverse Transcription Kit (Qiagen, Hilden, Germany) according to the manufacturer’s instructions. Relative gene expression was quantified with SYBR green-based detection (Alkali Scientific) using a light cycler96 system (Roche) according to the manufacturer’s protocols. Relative gene expression was normalized to 18S mRNA using the 2^−ΔCt^ method. Fold induction of gene expression was normalized to baseline expression in uninfected mice using the 2^−ΔΔCt^ method.

### Abs and Flow Cytometric Analysis

Ab cell staining was performed as previously described (17). For extracellular staining, cells were washed in PBS then stained with Live Dead Fixable Aqua (Life Technologies) for 30 min. Cells were stained with extracellular antibodies (see below), washed, and fixed in 2% formaldehyde. For intracellular staining, the cells were stimulated with 50 ng/ml PMA and 1 μg/ml ionomycin (Millipore) for 6 h (lung and LALN leukocytes) or heat-killed *C. neoformans* for 24 h (spleen cells) prior to Ab staining. Brefeldin A and monensin (BioLegend) were added for the last 4 h. The cells were harvested, stained with extracellular, and subsequently intracellular antibodies (see below). Data were collected on a FACS LSR2 flow cytometer using FACSDiva software (Becton Dickinson Immunocytometry Systems, Mountain View, CA, USA) and analyzed using FlowJo software (Tree Star, San Carlos, CA, USA).

The following gating strategy was used to identify leukocyte subsets in the lungs. First, consecutive gates identified singlets, live cells and CD45^+^ leukocytes. Next, a series of selective gates were used to identify neutrophils (CD11b^+^Ly6G^+^); eosinophils (SSC^high^CD11c^low^/SiglecF^+^); Alveolar macrophages (CD11c^high^/SiglecF^+^); and Ly6C^high^ monocytes (CD11c^−^/CD11b^+^/Ly6C^high^). In cells expressing both CD11c and high levels of MHCII, autofluorescent (AF^+^) exudate macrophages were distinguished from non-AF (AF^−^) DCs. Thereafter, DC were further separated into moDC, CD11b^+^ DC and CD103^+^ DC as follows: moDC were gated as CD11c^+^MHCII^high^CD64^+^ cells, then remaining CD11c^+^MHCII^high^CD64^−^ cells were further divided into CD11b^+^ DC and CD103^+^ DC based on the expression of SIRPα and XCR1, respectively (31). A similar gating strategy was used for leukocytes in the LALN. To identify CD4^+^ Th cells, singlet and live cells were first selected and the total numbers of CD45^+^ leukocytes were identified. Next, the CD3^+^CD4^+^ cells were identified and the expression levels of specific cytokines (IFN-γ, TNF-α, IL-5, and IL-13) or transcriptional factors (Gata3) were evaluated. Total numbers of each cell population were calculated by multiplying the frequency of the population by the total number of leukocytes (the original hemocytometer count of total cells). Isotype control antibodies were used to set gates for positive events in all flow cytometric analyses.

### Calculations and Statistics

All values are reported as mean ± SEM. Unpaired non-parametric test (Mann–Whitney) or two-way ANOVA with a Bonferroni *post hoc* test were used for comparisons of individual means. Statistical calculations were performed using GraphPad Prism version 6.00 (GraphPad Software, San Diego, CA, USA). Means with *p* values <0.05 were considered significantly different.

## Results

### MARCO Expression Promotes Fungal Persistence and Systemic Dissemination during the Efferent Phase of *C. neoformans* Infection

**Figure 1:**
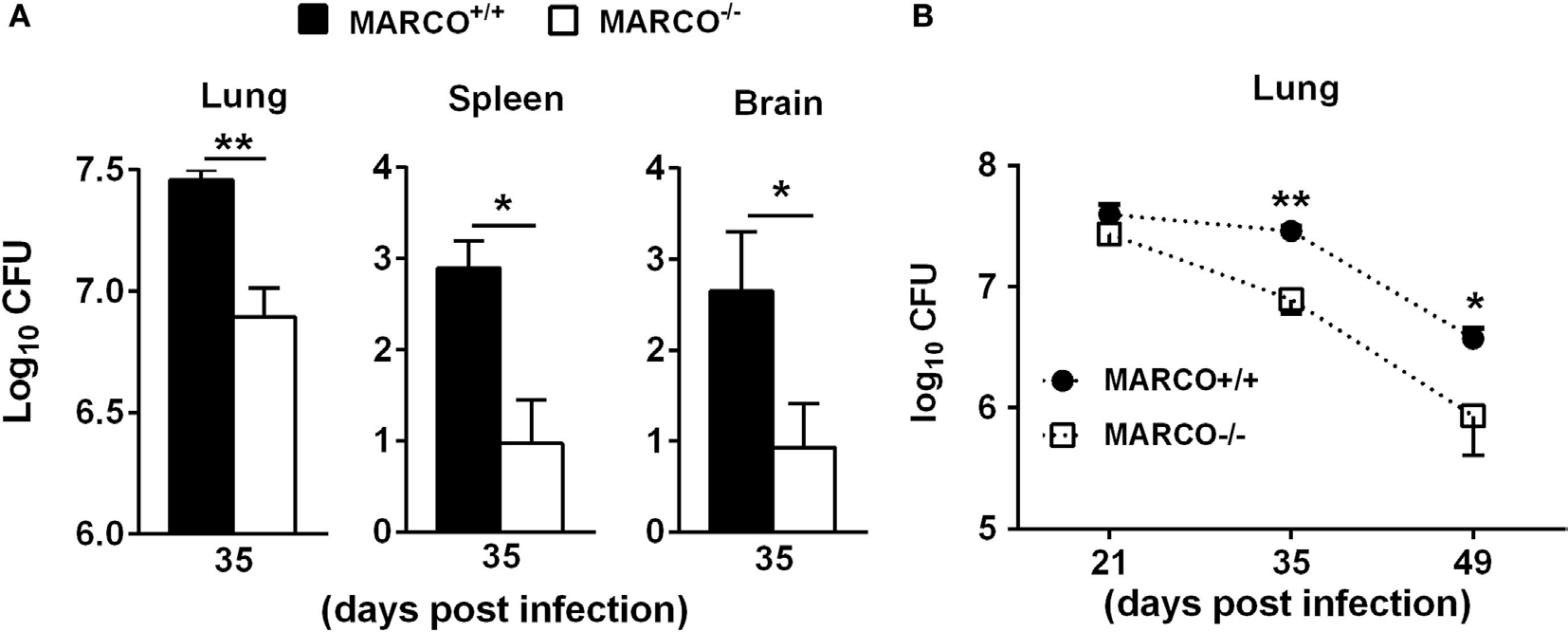
Macrophage receptor with collagenous structure (MARCO) expression contributes to fungal growth and dissemination during the efferent phase of *Cryptococcus neoformans* infection. MARCO^+/+^ and MARCO^−/−^ mice were infected intratracheally with 10^4^ *C. neoformans* 52D. **(A)** MARCO expression impaired fungal clearance in the lungs, spleens, and brains at 35 dpi during *C. neoformans* infection. **(B)** Kinetics of pulmonary fungal clearance in the MARCO^+/+^ and MARCO^−/−^ mice during efferent phase of *C. neoformans* infection. Results represent mean ± SEM from one of two separate matched experiments (*n* = 5 mice for each time point). *p < 0.05, **p < 0.01.

Our previous study showed that MARCO contributes to fungal control during the early/afferent phase of *C. neoformans* infection (19). However, whether MARCO expression affects long-term adaptive immunity against *C. neoformans* is unclear. To explore the function of MARCO during the late/efferent phase of cryptococcal infection, we infected WT and MARCO^−/−^ mice with *C. neoformans* strain 52D and analyzed the fungal burden at 35 days post infection (dpi). Whereas our prior results demonstrated that MARCO-deficient mice have increased fungal burden at the early stage of infection (19), we found that MARCO-deficient mice exhibit decreased pulmonary fungal burden at 35 dpi compared to WT mice (Figure 1A). Fungal burdens in spleen and brain were also lower in MARCO^−/−^ mice compared to WT mice at 35 dpi (Figure 1A). We further evaluated the effects of MARCO on the kinetics of pulmonary fungal growth during the efferent phase of *C. neoformans* infection. While equivalent growth of yeast in the lungs was noted in WT and MARCO^−/−^ mice at 21 dpi, MARCO deficiency resulted in lower fungal burden at 35 and 49 dpi (Figure 1B). Together, our results demonstrate that MARCO expression promotes pulmonary fungal persistence and systemic dissemination during the efferent phase of *C. neoformans* infection.

### MARCO Expression Promotes Non-Protective Th2 Response in the Lungs during the Efferent Phase of *C. neoformans* Infection

T helper cell polarization in the lungs is strongly correlated with clearance and persistence during the efferent phase of *C. neoformans* infection (32). To determine how MARCO expression affects T cell-mediated responses in the lungs, we analyzed leukocyte populations in the infected lungs of WT and MARCO^−/−^ mice using flow cytometry. The accumulation of total leukocytes and leukocyte subsets, including eosinophils, alveolar macrophages, and total DCs, in the infected lungs were similar between WT and MARCO^−/−^ mice at 21, 35, and 49 dpi (Figure S1 in Supplementary Material).

**Figure 2:**
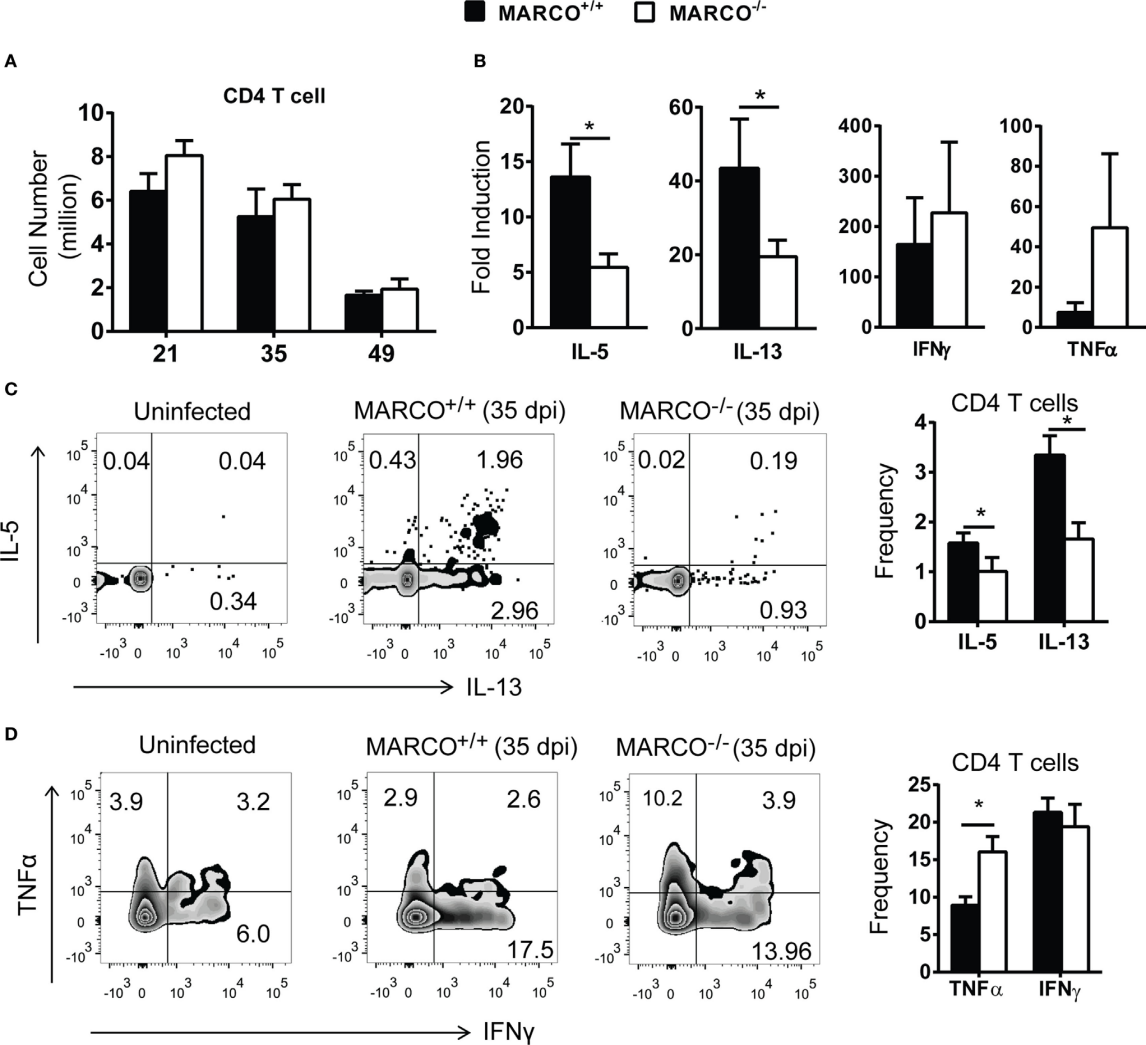
Macrophage receptor with collagenous structure (MARCO) expression promotes Th2 response in the lungs during the efferent phase of *Cryptococcus neoformans* infection. **(A)** MARCO is dispensable for the accumulation of CD4^+^ T cells in the lungs of *C. neoformans*-infected mice. **(B)** MARCO expression facilitates *IL-5* and *IL-13* but not *IFN-*γ and *TNF-*α mRNA expression by total lung leukocytes at 35 dpi during *C. neoformans* infection. **(C)** MARCO expression promotes IL-5 and IL-13 production by CD4^+^ T cells in the lungs of *C. neoformans*-infected mice. **(D)** MARCO expression inhibits TNF-α production by CD4^+^ T cells in the lungs of *C. neoformans*-infected mice. Results represent mean ± SEM (*n* = 5) from one of at least two independent experiments. *p < 0.05.

We next examined the effect of MARCO on T cell polarization during *C. neoformans* infection. There was no difference in the accumulation of CD4^+^ T cells in the lungs of *C. neoformans*-infected MARCO^−/−^ and WT mice during the efferent phase of cryptococcal infection (Figure 2A). However, MARCO deficiency resulted in reduced *Il-5* and *Il-13* mRNA expression, but not *IFN-*γ and *TNF-*α mRNA expression, by total lung leukocytes isolated from infected mice at 35 dpi, suggesting an impaired Th2 response in MARCO-deficient mice (Figure 2B). Phenotypes of T helper cells were further analyzed by intracellular staining using flow cytometry. Frequencies of CD4 T cells expressing IL-5 and IL-13 were significantly lower in MARCO^−/−^ mice compared to WT mice at 35 dpi (Figure 2C). By contrast, MARCO deficiency led to higher frequencies of CD4^+^ T cells expressing TNF-α albeit without affecting those expressing INF-γ at 35 dpi (Figure 2D). Collectively, our data demonstrated that MARCO expression did not affect accumulation of lung myeloid or lymphoid cells but altered the balance of T cell polarization away from protective Th1 responses toward non-protective Th2 responses in the lungs during the efferent phase of *C. neoformans* infection.

### MARCO Expression Interferes with Classical Activation of Pulmonary Exudate Macrophages during the Efferent Phase of *C. neoformans* Infection

**Figure 3:**
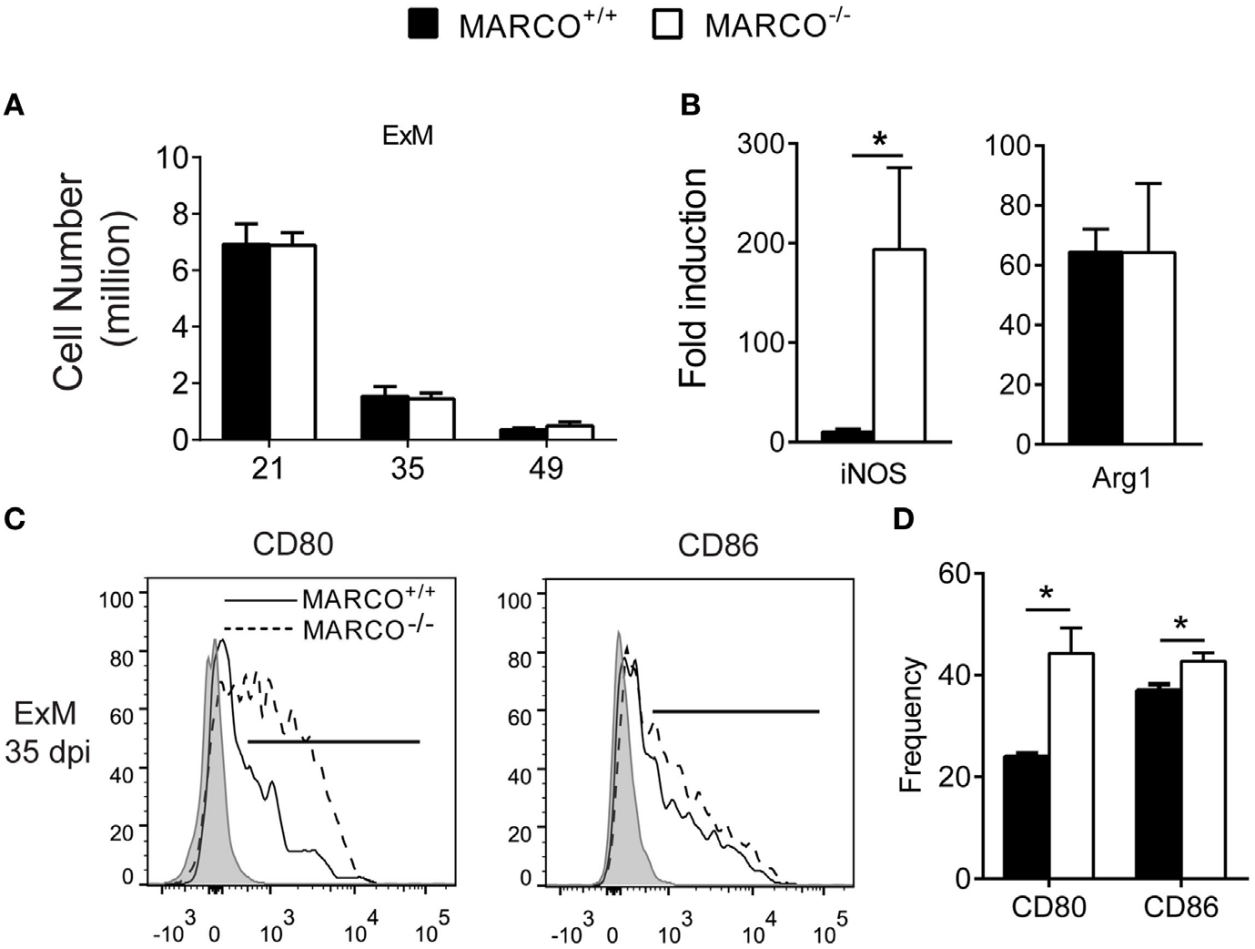
Macrophage receptor with collagenous structure (MARCO) expression impairs classical activation of macrophages in the lungs during the efferent phase of *Cryptococcus neoformans* infection. **(A)** Accumulation of exudate macrophages was not affected by MARCO deletion during *C. neoformans* infection. **(B)** MARCO impairs iNOS expression by adherent macrophages from lungs of infected mice at 35 dpi. **(C,D)** MARCO impairs expression of CD80 and CD86 by lung exudate macrophages at 35 dpi. Results represent mean ± SEM (*n* = 5) from one of two independent experiments. *p < 0.05. ExM, exudate macrophages.

The balance of Th1/Th2 cytokines influences macrophage activation status and their subsequent fungicidal activities (33, 34). Exudate macrophages derived from recruited monocytes during infection have been shown to be the most efficient effector cells in killing *C. neoformans* (35). We next sought to determine whether MARCO expression affects the activation status of pulmonary macrophages during the efferent phase of *C. neoformans* infection. Equivalent recruitment of exudate macrophages was noted between infected MARCO^−/−^ and WT mice during the efferent phase of *C. neoformans* infection (Figure 3A). However, MARCO deficiency resulted in augmented mRNA expression of *iNOS* but not *Arg1* by lung macrophages at 35 dpi (Figure 3B). Moreover, we found that MARCO deficiency resulted in significantly increased expression of CD80 and CD86 by exudate macrophages at 35 dpi (Figures 3C,D), consistent with improved pulmonary fungal clearance in these mice. Interestingly, expression of CD80, CD86, iNOS, and Arg1 by alveolar macrophages was similar between infected MARCO^−/−^ and WT mice (not shown). Thus, consistent with the diminished efferent Th1 responses, MARCO expression is associated with impaired classical activation of exudate macrophages in the lungs during the efferent phase of *C. neoformans* infection.

### MARCO Expression Enhances Non-Protective and Opposes Protective Systemic Responses during the Efferent Phase of *C. neoformans* Infection

**Figure 4:**
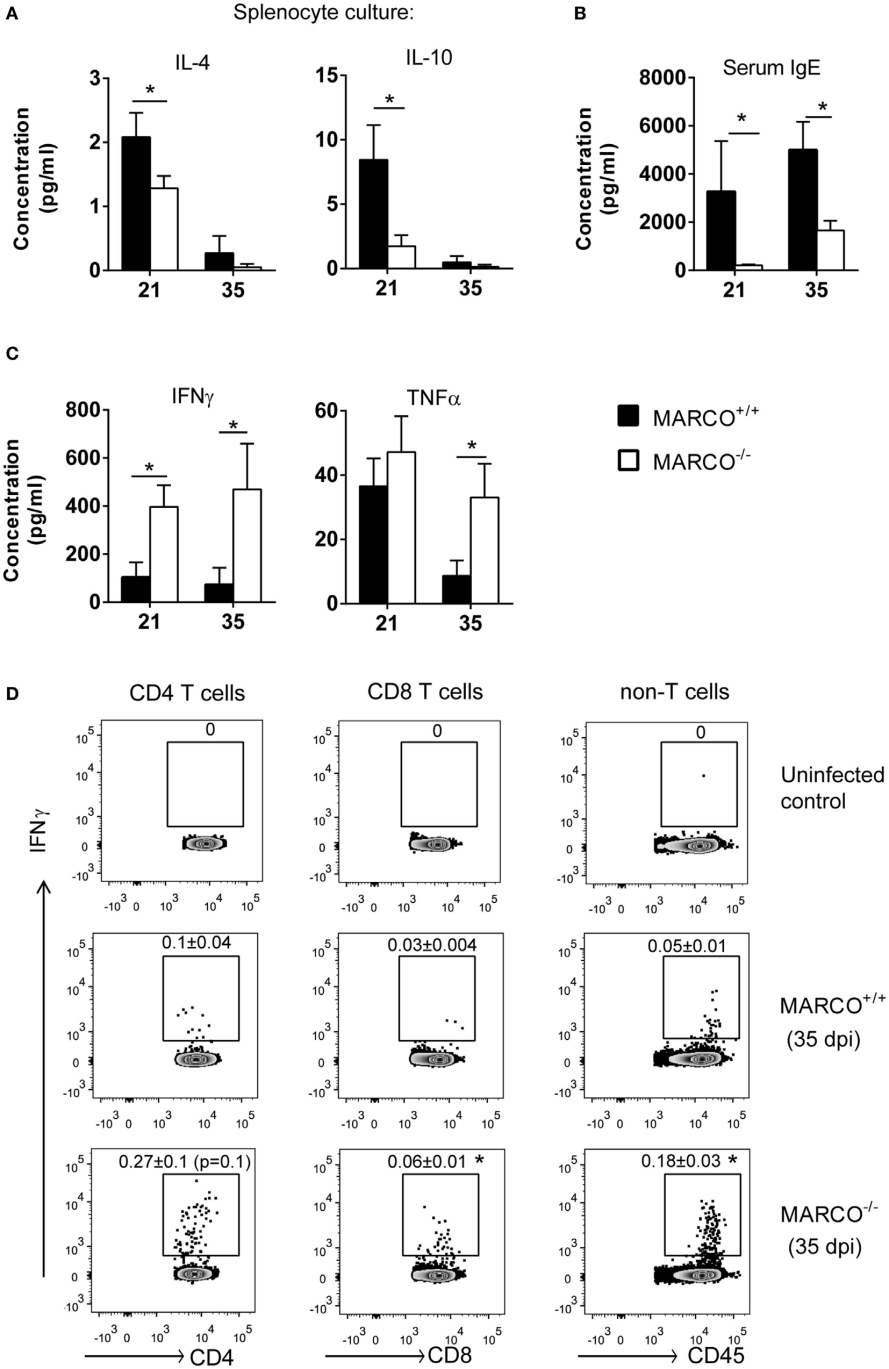
Macrophage receptor with collagenous structure (MARCO) expression promotes systemic Th2 response and inhibits Th1 response during the efferent phase of *Cryptococcus neoformans* infection. Spleen leukocytes were isolated from infected MARCO^−/−^ and MARCO^+/+^ mice at 21 and 35 dpi. After stimulated with heat-killed *C. neoformans* for 48 h, cytokine production in the supernatant of cell cultures was analyzed by cytometric bead assay. **(A)** MARCO expression increases IL-4 and IL-10 production by spleen leukocytes at 21 dpi. **(B)** MARCO expression significantly increases serum IgE level in mice infected with *C. neoformans*. **(C)** MARCO expression inhibits production of IFN-γ and TNF-α by spleen leukocytes at 21 or 35 dpi. **(D)** MARCO expression inhibits production of IFN-γ by CD4^+^ T cells, CD8^+^ T cells and non-T cells (CD4^−^CD8^−^) at 35 dpi. Splenocytes were stimulated with heat-killed *C. neoformans* and analyzed by intracellular flow cytometry. Results represent mean ± SEM (*n* > 4). *p < 0.05.

Since MARCO affected extra-pulmonary *C. neoformans* dissemination, we next studied its effects on systemic immune responses during the efferent phase of cryptococcal infection. To accomplish this, we evaluated the cytokine production by splenocytes of infected MARCO^−/−^ and WT mice in response to cryptococcal antigen. Consistent with results from the lungs, production of non-protective cytokines, including IL-4 and IL-10, was lower by splenocytes from MARCO^−/−^ compared to WT mice at 21 dpi (Figure 4A). The concentration of serum IgE, a hallmark of systemic Th2 response, was also significantly lower in MARCO^−/−^ mice compared to WT mice at 21 and 35 dpi (Figure 4B). Furthermore, MARCO deficiency led to elevated production of beneficial Th1 cytokines such as IFN-γ (21 and 35 dpi) and TNF-α (35 dpi) by splenocytes in response to cryptococcal antigen stimulation (Figure 4C). We next investigated whether T cells were the major source of antigen-triggered IFN-γ production in splenocyte cultures using intracellular flow cytometric analysis. We identified increased frequencies of IFN-γ-producing CD8^+^ T cells in MARCO^−/−^ mice compared to WT mice (Figure 4D). We also observed a similar, strong trend in increased frequencies of IFN-γ-producing CD4^+^ T cells in MARCO^−/−^ mice compared to WT mice (*p* = 0.1) (Figure 4D), which became significant when splenocyte culture were stimulated with PMA and ionomycin (data not shown). Interestingly, we also found a higher frequency of non-T cell populations (CD4^−^CD8^−^) producing IFN-γ in antigen stimulated splenocyte cultures from MARCO^−/−^ mice compared to those from WT mice (Figure 4D). These results indicate that MARCO expression also broadly affects IFN-γ production by cells outside the T cell compartment. Collectively, we found that MARCO expression promotes a systemic shift away from protective responses toward non-protective responses during the efferent phase of *C. neoformans* infection.

### MARCO Expression Contributes to Th2 Priming in the LALNs during *C. neoformans* Infection

**Figure 5:**
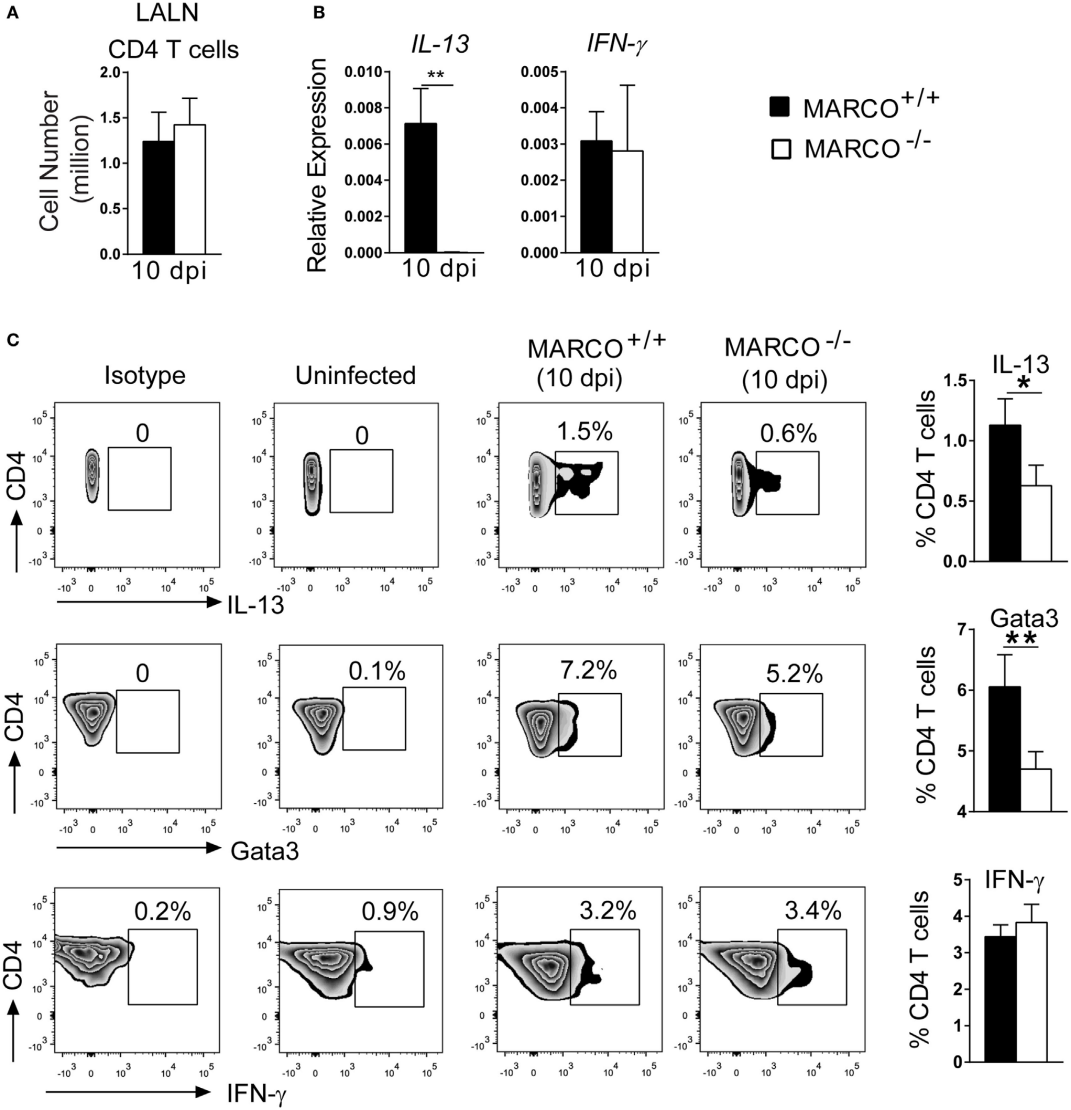
Macrophage receptor with collagenous structure (MARCO) promotes Th2 response in the LALN of *Cryptococcus neoformans*-infected mice. **(A)** MARCO is dispensable for accumulation of CD4^+^ T cells in the LALN of *C. neoformans*-infected mice at 10 dpi. **(B)** Expression of IL-13 and IFN-γ by total LALN leukocytes from infected MARCO^−/−^ and MARCO^+/+^ mice at 10 dpi were analyzed by RT-qPCR. Note that MARCO enhanced mRNA expression of IL-13 but not IFN-γ by total LALN leukocytes. Expression of each target gene was normalized to the 18S housekeeping gene. **(C)** MARCO expression promotes IL-13 and Gata3 but not IFN-γ protein production by CD4^+^ T cells in the LALN of *C. neoformans*-infected mice. Results represent mean ± SEM (*n* = 5) with similar results from two independent experiments. *p < 0.05.

We next dissected the mechanisms by which MARCO modulates Th polarization. T cells are primed in the draining lymph nodes and their polarization is orchestrated by distinct DC subsets during *C. neoformans* infection (36). We, thus, looked at the priming of T cells in the LALN at 10 dpi, an early time point at the onset of T cell polarization. Though MARCO deficiency had no effect on the T cell expansion in LALNs at 10 dpi (Figure 5A), we found reduced expression of *IL-13* by the LALN leukocytes of infected MARCO^−/−^ mice compared to WT mice (Figure 5B). Furthermore, expression of *IFN-*γ and the transcription factors *T-bet* and *Eomesodermin* by LALN leukocytes were similar between MARCO^−/−^ and WT mice (Figure 5B and data not shown). To further assess whether MARCO promotes Th2 priming in the LALN during *C. neoformans* infection, we performed intracellular staining for cytokines and transcription factors in LALN leukocytes. Consistent with our gene expression analysis, we found that MARCO deficiency resulted in reduced frequencies of LALN CD4^+^ T cells expressing Th2-associated cytokine IL-13 and transcriptional factor Gata3 at 10 dpi (Figure 5C). By contrast, MARCO deficiency had no effect on IFN-γ expression by CD4^+^ T cells in the LALN at 10 dpi. Thus, MARCO expression enhances early Th2 priming but appears to have little or no effect on early Th1 priming in the LALN during *C. neoformans* infection.

### MARCO Expression Promotes Accumulation of CD11b^+^ cDC in the LALN during *C. neoformans* Infection

**Figure 6:**
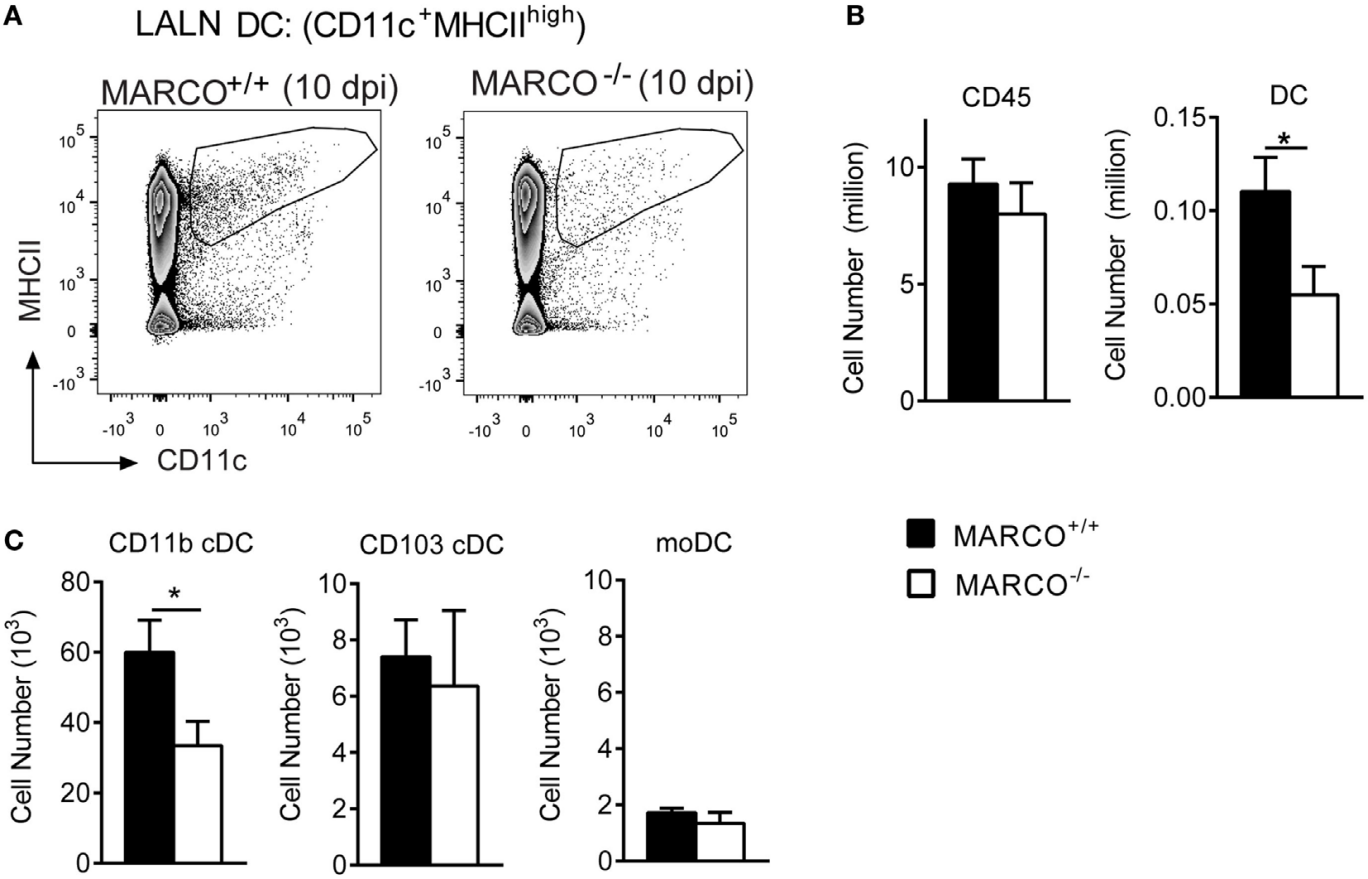
Macrophage receptor with collagenous structure (MARCO) expression promotes the accumulation of CD11b^+^ cDC in the lung-associated lymph node (LALN) of *Cryptococcus neoformans*-infected mice. **(A,B)** MARCO expression promotes the accumulation of dendritic cell (DC) but not total leukocytes in LALN of *C. neoformans*-infected mice at 10 dpi. **(C)** MARCO expression promotes the accumulation of CD11b^+^ cDC but not CD103^+^ cDC and moDC. Results represent mean ± SEM (*n* = 5), with similar results from two independent experiments. *p < 0.05. cDC, conventional dendritic cells; moDC, monocytes-derived dendritic cells.

Pulmonary CD11b^+^ cDC have been shown to orchestrate the development of non-protective Th2 responses during cryptococcal infection (15). To test whether MARCO expression affects the accumulation of DC in the LALN, we analyzed leukocyte populations in the LALN of infected MARCO^−/−^ and WT mice at 10 dpi. While total leukocyte accumulation in the LALN was not altered in MARCO-deficient mice, MARCO expression significantly promoted the accumulation of migratory DC (CD11c^+^MHCII^high^) in the LALN of infected mice (Figures 6A,B). We further explored whether MARCO affected the accumulation of specific DC subsets in the LALN during *C. neoformans* infection. Notably, MARCO deficiency selectively impaired accumulation of CD11b^+^ cDC, but had no effect on the accumulation of CD103^+^ cDC and moDC subsets in the LALN of *C. neoformans*-infected mice (Figure 6C). Thus, MARCO expression is critical for the accumulation of CD11b^+^ cDC in the LALN during *C. neoformans* infection.

### MARCO Expression Promotes Alternative Activation and Inhibits Classical Activation of CD11b^+^ cDC in LALNs during *C. neoformans* Infection

**Figure 7:**
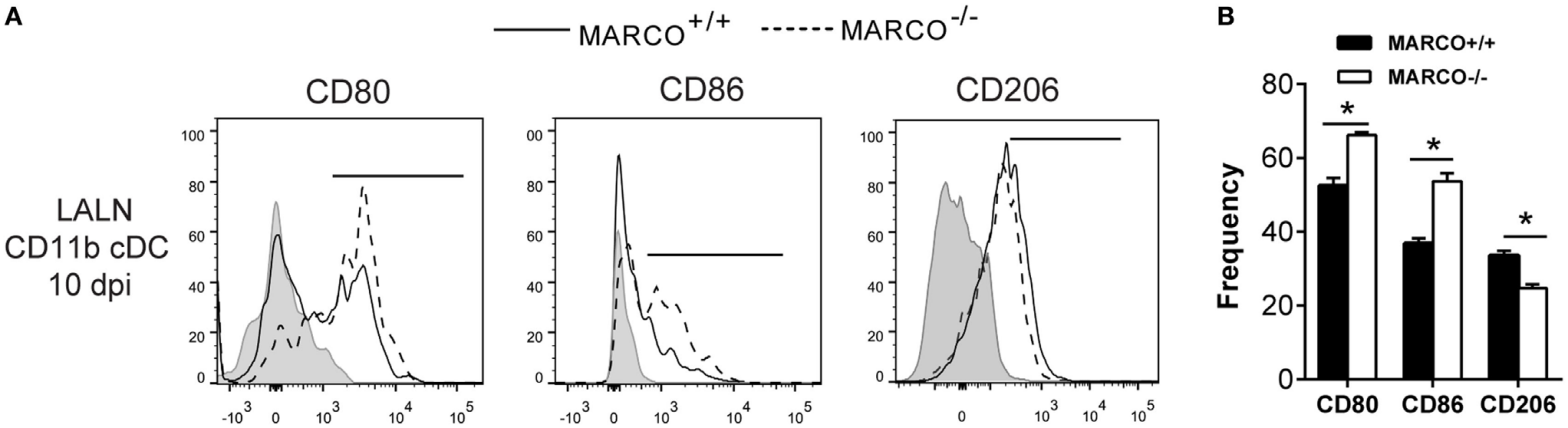
Macrophage receptor with collagenous structure (MARCO) expression promotes alternative activation and inhibits classical activation of CD11b^+^ conventional DC (cDC) in the lung-associated lymph node (LALN) of *Cryptococcus neoformans*-infected mice. **(A)** Surface expression of CD80, CD86, and CD206 by CD11b^+^ cDC was analyzed by flow cytometry. **(B)** Bar graphs represent percentage of CD11b^+^ cDC expressing CD80, CD86, and CD206. Note that CD11b^+^ cDC from infected MARCO^+/+^ mice exhibited higher expression of CD80 and CD86 but lower levels of CD206 compared to CD11b^+^ cDC from infected MARCO^−/−^ mice. Results represent mean ± SEM (*n* = 5), with similar results from two independent experiments. *p < 0.05.

**Figure 8:**
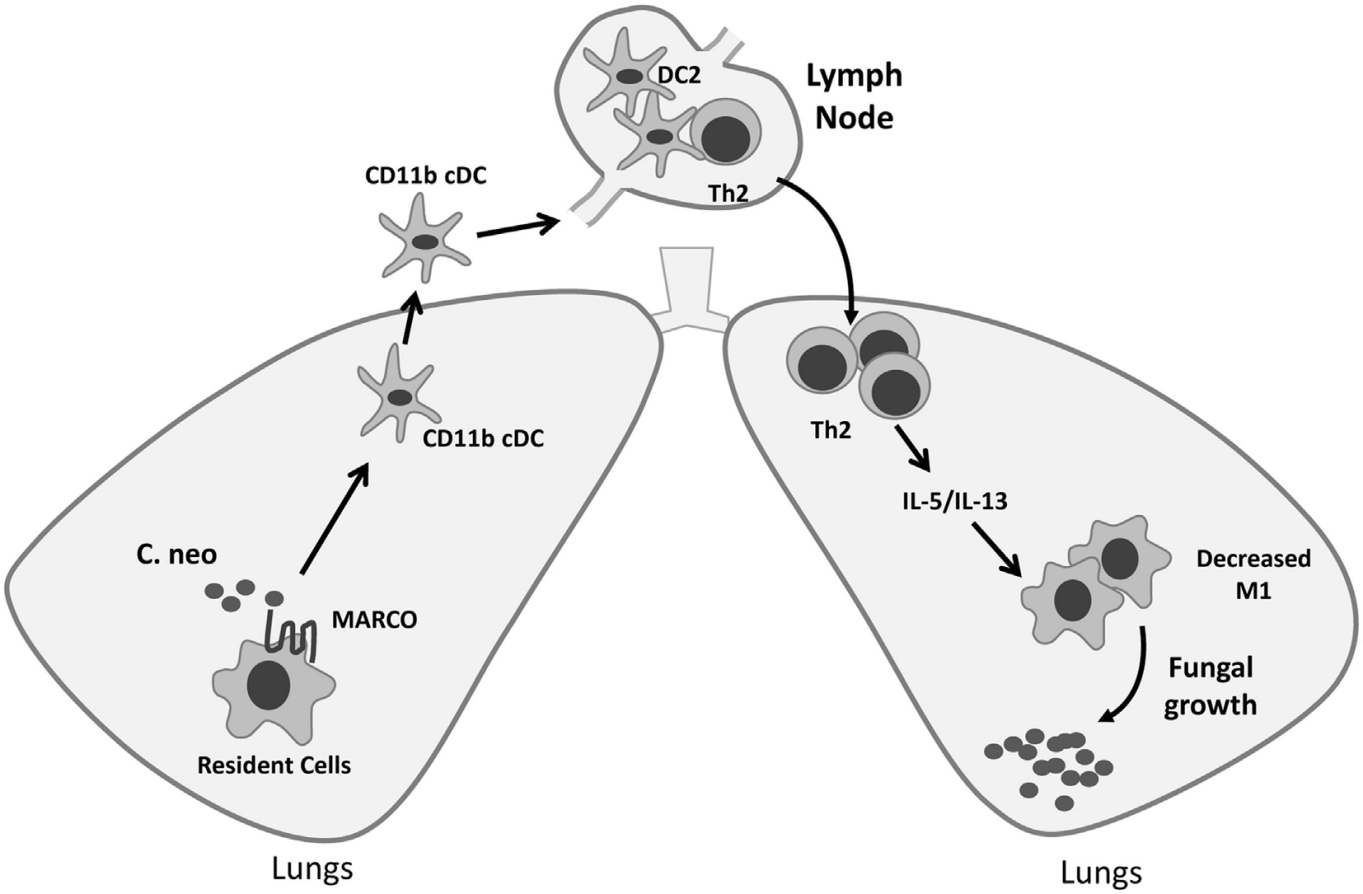
Roles of macrophage receptor with collagenous structure (MARCO) in orchestrating adaptive immunity during fungal infection. MARCO expression contributes to accumulation of CD11b^+^ conventional DC (cDC) and their alternative activation in the lung-associated lymph node (LALN), which promotes the development of Th2 responses in the LALN and lungs during *Cryptococcus neoformans* infection. Thus, efferent control of fungal growth was impaired along with increased production of type 2 cytokines IL-5 and IL-13, and alternative activation of effector cells in the lungs of MARCO^+/+^ mice.

The activation status of DC in the LALN can be influenced by fungal virulence factors and plays a vital role in the control of T cell polarization (16, 37). We further assessed the effect of MARCO on the activation status of CD11b^+^ cDC in the LALN during *C. neoformans* infection by analyzing the expression of classical activation markers (CD80 and CD86) as well as alternative activation marker (CD206) on CD11b^+^ cDC. We found that MARCO deficiency led to increased expression of CD80 and CD86 and decreased expression of CD206 by CD11b^+^ cDC in the LALN at 10 dpi (Figures 7A,B). Interestingly, MARCO deficiency had no effect on the activation status of CD103^+^ cDC and moDC in LALN of infected mice (not shown). These data show that MARCO expression specifically promotes alternative activation and inhibits classical activation of CD11b^+^ cDC in the LALN. Collectively, this MARCO-dependent accumulation and alternative activation of CD11b^+^ cDC in the LALN may represent a critical mechanism through which MARCO promotes the development of a stronger Th2 response and downregulates the Th1 response, leading to fungal persistence during the efferent phase of *C. neoformans* infection (Figure 8).

## Discussion

Although our previous study demonstrated that scavenger receptor MARCO is involved in the phagocytosis of *C. neoformans* by lung leukocytes and early recruitment of myeloid cells, the function of MARCO in the later stages of adaptive antifungal immunity remains unknown. Here, we demonstrated that MARCO expression ultimately promotes fungal persistence and dissemination by directly or indirectly contributing to the development of non-protective responses during cryptococcal infection. Our immunological analysis mechanistically linked these effects during the adaptive phase with increased pulmonary and systemic Th2 responses and impaired classical activation of macrophages in the lungs. We further show that the upstream accumulation and alternative activation of CD11b^+^ cDC in LALN of *C. neoformans*-infected mice provides a likely mechanism through which *C. neoformans* exploits MARCO to trigger a shift to Th2 responses. Collectively, our study uncovers novel cellular and molecular pathway through which *C. neoformans* evades host defenses and induces non-protective antifungal immunomodulation.

Our current study revealed the novel observation that MARCO expression significantly promotes fungal persistence and extra-pulmonary dissemination during the efferent phase of *C. neoformans* infection. Interestingly, our previous study demonstrated that MARCO orchestrates innate defenses and contributes to fungal containment during initial response to cryptococcal infection by promoting early inflammatory cytokine production in the lungs and phagocytosis by myeloid cells (19). These results show that, in the grand scheme, the initial benefits of MARCO expression during the innate phase were gradually replaced by the non-protective effects induced by MARCO during the efferent phase of *C. neoformans* infection. Collectively, we found that MARCO can play distinct functions in host defenses to pathogens, protective during the relatively short innate phase and then non-protective during subsequent efferent phase.

Distinct roles of specific factors in innate versus adaptive responses, while rare, are not unique to MARCO and can be found in other immune receptors, such as T-cell immunoglobulin domain and mucin domain 3 (TIM3), which was shown to promote pro-inflammatory innate responses while restraining Th1 responses (38). While it is not uncommon for pathogens to exploit loopholes in the signaling cascades of the immune system and hijack protective pathways, our results showing dual roles of MARCO during cryptococcal infection may provide insights that explain why different genetic variants of MARCO differentially affect health of the human population. In human pulmonary tuberculosis, some genetic variants of MARCO are associated with susceptibility, while other variants are associated with resistance (39). Given the similarities of pathogenicity between *C. neoformans* and *M. tuberculosis* (both can survive and grow intracellularly in the alveolar macrophages and require Th1-cell help for their eradication), it is possible that these distinct variants may affect the magnitude of protective versus non-protective effects of MARCO. Another element of pathogenesis shared by *C. neoformans* and *M. tuberculosis* is their ability to induce latent infections (40, 41). Since, non-protective elements of the immune response contribute to cryptococcal persistence (42, 43), MARCO and other factors exploited by *C. neoformans* may in fact contribute to the development of cryptococcal latency. Thus, possible contribution of MARCO expression to long-term latency of *C. neoformans* infection and whether genetic variants of MARCO are associated with susceptibility to cryptococcosis in humans warrants further study.

The effects of MARCO in the development of adaptive immunity were less well explored compared to its well-studied innate functions in control of infections such as *Mycobacterium tuberculosis, Streptococcus pneumonia, and Leishmania major* (21–23). In this study, we showed that MARCO expression can be later exploited by *C. neoformans* to modulate T cell polarization during the efferent phase of infection. Consistent with the higher fungal burden in WT mice, MARCO expression led to enhancement of non-protective Th2 responses, diminished protective Th1 responses, and impaired classical activation of macrophages in the lungs during the efferent phase of *C. neoformans* infection (Figure 8). Interestingly, while the effects of MARCO on induction of systemic and pulmonary Th2 bias were similar (Th2 cytokine production in lungs and spleen and corresponding changes in serum IgE level), divergent functions of MARCO were found when it comes to IFN-γ production. MARCO expression was associated with diminished expression of IFN-γ by both T cells and non-T cells in the spleens, while we did not observe any effects of MARCO on IFN-γ production by the lung and LALN cells. Thus, while our current study substantially advances our understanding of the role of MARCO as an important modulator of host adaptive immunity during fungal infections, future studies are needed to fully characterize the mechanisms by which MARCO plays divergent roles in different organs of the infected host.

Molecular and cellular mechanisms of non-protective Th2 response development during *C. neoformans* infection are incompletely understood. Pathogen virulence factors, such as capsule, chitin, urease, and other lipid mediators, promote Th2 responses during *C. neoformans* infection (8, 44, 45), however, host signaling pathways exploited by fungal pathogens that contribute to Th2 development are less studied. Though critical roles of pulmonary CD11b^+^ cDC in Th2 response development during *C. neoformans* infection was demonstrated (15), the underlying mechanisms are not fully elucidated. In our study, we demonstrated that the upstream Th2 priming in the draining lymph nodes was enhanced by MARCO expression. More importantly, our results showed that the accumulation and alternative activation of CD11b^+^ cDC also depended on MARCO expression. Thus, the increased accumulation and alternative activation of CD11b^+^ cDC is likely a crucial mechanism by which *C. neoformans* exploits MARCO to promote the Th2 response and downregulate Th1 response during infection. However, at this time, we cannot rule out that other possible cell subsets affected by *C. neoformans* in a MARCO-dependent fashion might also enhance non-protective Th2 responses.

The current study demonstrates that MARCO exhibits some interesting similarities and differences during fungal infections when compared with our previous study on the role of another scavenger receptor, SR-A (17). While our studies demonstrated that *C. neoformans* can exploit multiple scavenger receptors to facilitate its survival in the host by triggering the same type of immunomodulation, there are significant differences between the effects of SR-A and MARCO on DC activation. SR-A promotes the early production of type 2 cytokines in both the lungs and LALN during the afferent phase of *C. neoformans* infection and globally contributed to the alternative activation of DC at both sites (17). By contrast, MARCO contributes to increased early IFN-γ production and classical activation of moDC in the lungs (19), at the same time selectively enhances the accumulation and alternative activation of CD11b^+^cDC in the LALN of *C. neoformans*-infected mice. These seemingly discrepant effects within mucosal sites and regional lymph nodes demonstrate that MARCO signaling is likely contextual, depending on DC subsets and/or effects of local micro-environments. While future studies are needed to clarify this, our studies indicate that both MARCO and SR-A can promote Th2 polarization during cryptococcal infection possibly *via* different mechanisms.

In summary, our novel findings show that MARCO facilitates cryptococcal persistence in the host through promoting non-protective Th2 responses during the efferent phase of *C. neoformans* infection. We identify MARCO-dependent accumulation and alternative activation of CD11b^+^ cDC as one mechanism explaining these effects. Thus, this study substantially advances our understanding about the roles of MARCO in the adaptive phase of antifungal immunity.

## Ethics Statement

This study was carried out in accordance with the recommendations of University Committee on the Use and Care of Animals and the Veterans Administration Institutional Animal Care and Use Committee. All experiments and protocol were approved by the University Committee on the Use and Care of Animals and the Veterans Administration Institutional Animal Care and Use Committee.

## Author Contributions

JX and AF contributed to experimental design, experimental work, data analysis, and manuscript writing. LN, ZF, DC, and MI contributed to experimental work and manuscript editing. BBM, JC, and JO contributed to write and edit manuscript. MO contributed to secure funding, oversee the project, design framework, and experimental work.

## Conflict of Interest Statement

The authors declare that the research was conducted in the absence of any commercial or financial relationships that could be construed as a potential conflict of interest.
