# Supplementary material for: Exploitation of Scavenger Receptor, Macrophage Receptor with Collagenous Structure, by Cryptococcus neoformans Promotes Alternative Activation of Pulmonary Lymph Node CD11b+ Conventional Dendritic Cells and Non-Protective Th2 Bias
Source: Front Immunol. 2017 Sep 28;8:1231. doi: 10.3389/fimmu.2017.01231 (PMC5624996; doi:10.3389/fimmu.2017.01231)
Supplement: Supplementary file 1 [file Image_1.PDF]

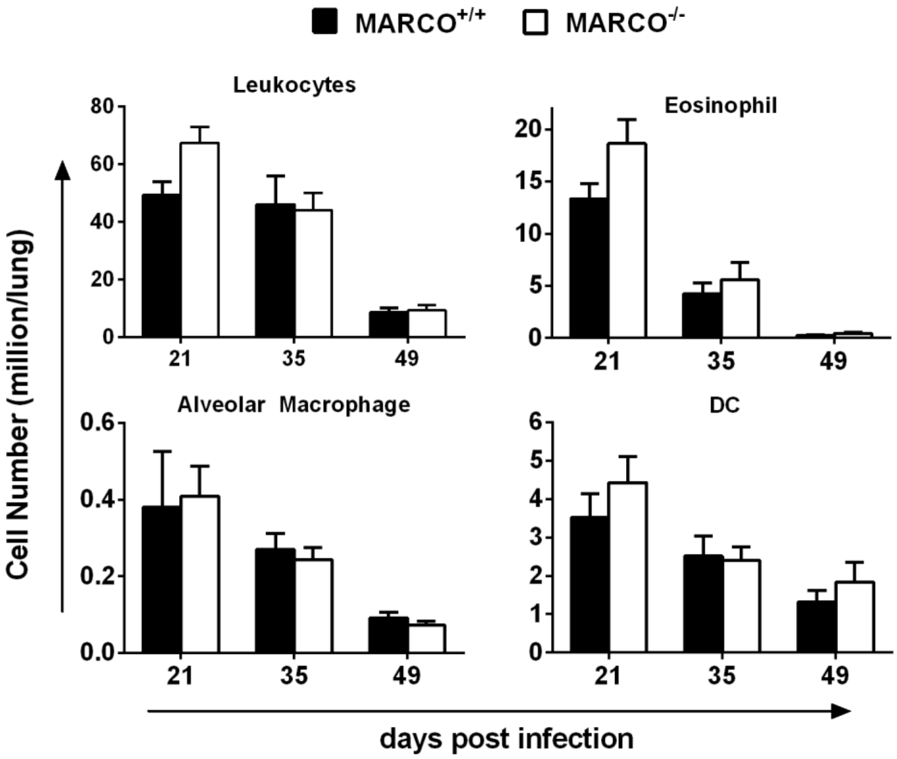

Fig. S1 MARCO expression has no effect on leukocyte recruitment during the efferent phase of *C. neoformans* infection. Lung leukocytes from infected MARCO$^{+/+}$ and MARCO$^{-/-}$ mice were isolated and analyzed by flow cytometry as per Material and Methods. Total numbers of leukocytes, eosinophils, alveolar macrophages, and DCs are shown. Note that no differences in cell recruitment exist between infected MARCO$^{-/-}$ and MARCO$^{+/+}$ mice. Results represent mean ± SEM (n = 5).
